# Contributions of distractor dwelling, skipping, and revisiting to age differences in visual search

**DOI:** 10.1038/s41598-024-83532-y

**Published:** 2025-01-13

**Authors:** Iris Wiegand, Mariska van Pouderoijen, Joukje M. Oosterman, Kay Deckers, Gernot Horstmann

**Affiliations:** 1 Donders Institute for Brain, Cognition and Behaviour, Radboud University, Postbus 9102, 6500 HC Nijmegen, The Netherlands; 2 Alzheimer Centrum Limburg, Department of Psychiatry and Neuropsychology, School for Mental Health and Neuroscience (MHeNs), Maastricht University, 6200 MD Maastricht, The Netherlands; 3 Department of Psychology, Bielefeld University, 33501 Bielefeld, Germany

**Keywords:** Cognitive ageing, Age-related slowing, Selective attention, Eye-movements, Distractor rejection, Target-distractor similarity, Psychology, Human behaviour

## Abstract

Visual search becomes slower with aging, particularly when targets are difficult to discriminate from distractors. Multiple distractor rejection processes may contribute independently to slower search times: dwelling on, skipping of, and revisiting of distractors, measurable by eye-tracking. The present study investigated how age affects each of the distractor rejection processes, and how these contribute to the final search times in difficult (inefficient) visual search. In a sample of Dutch healthy adults (19–85 years), we measured reaction times and eye-movements during a target present/absent visual search task, with varying target-distractor similarity and visual set size. We found that older age was associated with longer dwelling and more revisiting of distractors, while skipping was unaffected by age. This suggests that increased processing time and reduced visuo-spatial memory for visited distractor locations contribute to age-related decline in visual search. Furthermore, independently of age, dwelling and revisiting contributed stronger to search times than skipping of distractors. In conclusion, under conditions of poor guidance, dwelling and revisiting have a major contribution to search times and age-related slowing in difficult visual search, while skipping is largely negligible.

## Introduction

Visual search is a daily task that allows us locating a visually more or less distinctive target item surrounded by irrelevant distractors^1^. Visual search abilities decline with age^2^, which may cause difficulties with finding objects in cluttered environments as well as the quick and accurate identification of relevant visual information or landmarks. For example, detection of road signs, traffic signals, and potential hazards, is crucial for save navigation and driving in older age^3^. Similarly, locating specific information on computer screens is an ubiquitous part of any task that includes working with, and navigating through, digital interfaces, which becomes less efficient with increasing age^4^. While visual search is consistently found to be slower in older age, it is less clear whether age differences reflect deficits on specific visual processing stages or can be explained by generalized age-related slowing^5,6^. Slower search times in older age are particularly pronounced in difficult visual search tasks, in which the discriminability of the target is low due to high numbers of target-similar distractors^7–11^. Accordingly, it has been suggested that aging specifically affects filtering out distracting information^12,13^.

Notably, search times reflect the net outcome of multiple processes involved in visual search, which makes it difficult to identify and dissociate the processing stages from which age-related slowing may originate^14,15^. In the present study, we therefore use eye-movements as process-specific metrics of age differences in multiple distractor rejection processes that contribute to search times in a difficult visual search task: Dwelling on distractors, skipping of distractors, and revisiting of distractors^16–20^.

### Mechanisms of age differences in visual search: target guidance and distractor rejection

In laboratory visual search tasks, performance is typically measured as reaction time (RT) for correct decisions on whether a target is absent or present in displays that contain varying numbers of distractors. Search efficiency is quantified as the increase in RT as a function of the number of distractors in the search display, that is, the slope of the RT x set size function. Search efficiency is strongly influenced by the similarity between the target and distractors^21^. If a target is very dissimilar from its surrounding distractors, it “pops-out” and can be easily discriminated independently of the set size, resulting in search slopes near zero marking highly efficient search (i.e., adding distractors to the search array does not increase the search time for the target). With increasing target-distractor similarity, search becomes inefficient, reflected in steeper slopes, where the steepness of the search slope indicates the degree of inefficiency^22^. Besides general slowing, aging has little effects on efficient searches^23–25^. By contrast, older adults showed larger RT costs than younger adults with increasing inefficiency of visual search due to higher target-distractor similarity^7,11^.

Prominent guidance-based models of visual search, such as Guided Search (GS)^1,22,26^, explain search efficiency by the strength of attentional guidance to target locations in the visual field. According to GS, the visual field is represented in a spatiotopically organized activation map, where the amount of activation corresponds to the feature information matching a target template at each location, that is, evidence that a location contains the target. A target that is very dissimilar from the distractors, and matches the target template exclusively, results in a distinct peak in the activation map and a high signal-to-noise ratio. The high activation of the target location, and low activation of distractor locations, provides a strong guidance signal to attentionally select the target rapidly without attending to distractors, leading to short search times. The finding of similarly flat search slopes for efficient searches in younger and older age groups suggests that target guidance is largely preserved in older age^5,23,25^. Conversely, when targets and distractors share visual features, distractors partly match the target template and activity peaks occur also at distractor locations. Given the lower signal-to-noise ratio, the highest activation will not always be at the target location and multiple distractors may be attentionally selected before the target is finally found. Thus, if target-distractor similarity is very high, guidance towards the target is low, or may even be absent. Accordingly, older adults’ difficulties in such inefficient searches^7^ likely originate from distractor rejection processes that contribute to search performance besides target guidance. Recent eye-tracking studies in younger adults have highlighted the distinction of multiple distractor rejection processes in visual search, the *dwelling on distractors*, *skipping of distractors,* and *revisiting of distractors*^16,17,19,20^. However, adult age differences in these distinct distractor rejection processes have not been examined, yet.

In guidance-based search models, the primary mechanism to reject distractors is the *skipping of distractors*. With decreasing target guidance, more distractors will be attentionally selected and fewer distractors will be skipped. As an additional principle to guidance in target-absent trials, Chun and Wolfe (1996) suggested that distractor skipping depends on an adaptive quitting threshold that varies with guidance in target-present trials: An observer looking for a strongly guiding target may safely conclude that there is no target and skip most of the objects when they cannot locate the target with a single look. However, when a poorly guiding target is searched for, the observer may rather check all distractors before deciding that the target is absent^27,28^. Importantly, studies on age differences in perceptual decision making consistently show that older adults implement more conservative decision rules than younger adults^29^. Older adults were suggested to accumulate relatively more evidence and increase the decision threshold to compensate for higher sensory noise^30^. Similarly, the signal-to-noise ratio of the activation map may decrease with age, which increases the quitting threshold and lowers the skipping rates. Accordingly, in inefficient searches, where the rate of skipping is already low in younger age, skipping may be further reduced in older age, which contributes to prolonged search times.

Apart from the number of inspected distractors, also the *dwelling on distractors*, that is, the time needed to process each item, determines search speed^31^. Dwell times on distractors depend on the perceptual demands^31^ and increase with target-distractor similarity^32^, which is interpreted to reflect the processing requirements to distinguish the target from distractors, and directly contributes to the RT differences between easy and difficult conditions^16,17,19,20^. Dwell times were often reported to be longer in older adults, compared to younger adults^33^,^34^. This age-related increase in dwell times may result from sensory decline^2,35^, slowing of visual processing speed^36–38^, or the above mentioned need to accumulate more perceptual evidence before a decision is made to classify an object as a target or distractor^29^.

Finally, search (in)efficiency depends on the *revisiting of distractors*^39^. Ideally, a location in a search display should be visited only once. This requires some bookkeeping in memory, and failures of bookkeeping result in revisits. A key mechanisms assumed to prevent the inspection of previously searched locations is ‘Inhibition of return’ (IOR)^40,41^. IOR is thought to bias visual search toward novel locations by tagging and transient suppression of recently visited locations^42^. The capacity and duration of inhibitory tags is limited, thus, IOR may not fully prevent revisiting under search conditions where many distractors are scanned^43^. Alternatively, distractor revisiting may depend on visual memory for already visited locations^44^. Findings on age differences in revisiting are mixed. IOR is considered preserved, or even increased, with normal aging^45,46^. Accordingly, fewer revisits of previously examined distractor locations in older, compared to younger, adults, may be attributed to a stronger IOR in older age^47^. By contrast, the capacity of visual working memory is consistently found to be reduced in older age^36,48,49^. Accordingly, an increase of fixations in older age may indicate limited memory of the already inspected locations^50^.

### The present study

The aim of the present study was to investigate whether and how multiple, distinct distractor rejection processes—skipping, dwelling, and revisiting—are influenced by the observer’s age and to which degree they contribute to search times in inefficient visual search. We collected RT and eye-tracking data during a difficult visual search task in which target-distractor similarity was varied between blocks^16^. We used naturalistic stimulus material (faces) in which target-distractor similarity was manipulated by a distinct perceptual feature of the target (smiling with visible teeth)^32^. Specifically, observers searched for an emotional (happy) face target among neutral face distractors. The emotional targets were either closed-lipped, distractor-similar, faces or open-lipped, distractor dissimilar, faces. First, we examined age effects on skipping, dwelling, and revisiting. Second, we estimated the relative contributions of age, skipping, dwelling, and revisiting to RT. We expected that RT in this search task generally increase with age^7,51^. Furthermore, we expected that similarity and set size influence all distractor rejection processes, which contribute independently to RT. Finally, we hypothesize that the age-related slowing in RT can at least partly be explained by age differences in these distinct distractor rejection processes. Specifically, we expect that skipping decreases, while dwelling and revisiting increases with age.

## Methods

### Participants

We recruited 67 healthy participants of a broad adult age range (19–85 years) via existing databases and mouth-to-mouth advertisements, to participate in a neuropsychological test battery, of which 52 completed the eye-tracking experiment reported here. The test battery was administered by trained student test assistants. Participants were included in the study if they reported no prior history of psychiatric or neurological diseases and had adequate fluency in Dutch to understand the informed consent procedure, instructions, and questionnaires. Participants further underwent a visual and cognitive screening to make sure they were not color blind, had a (corrected) visual acuity of at least 20/25 based on the Snellen Chart, and/or showed no signs of (mild) cognitive impairment based on the Montreal Cognitive Assessment (MoCA^52^), indicated by scores > 24^53^. One participant was excluded due to a low MoCA score. Seven participants were excluded due to incomplete datasets, no recording of dwell times, and/or technical problems during the eye-tracking experiment. The exclusion of participants from the eye-tracking experiment was due to technical difficulties and unrelated to the participants’ abilities or age. The final sample consisted of 45 participants.

Notably, the trial-based analyses are based on large numbers of observations (see below). Thus, this study was well-powered to detect effects of dwelling, skipping, and revisiting on RT. Based on the previous results^16,17^, main effects of similarity, target presence, and set size on RT and eye-tracking measures are expected to be large. The sample size of 45 was also sufficient to detect a moderate main effect of age on dwelling, skipping, revisiting, or RT with a power of 0.80 (as there is no established procedure to determine power for multi-level linear models, we used simple bivariate correlation in the power analysis).

Demographic information of the final sample included in the study is reported in Table 1. The sample characteristics are reported separately for male and female participants and for participants who were in- and excluded in the supplementary online material (https://osf.io/2vc4t/). Importantly, age did not systematically vary with the educational level, as indicated by small and insignificant correlations between age and the years of education (r = − 0.06) and scores in the Dutch adult reading test (Nederlandse Leestest voor Volwassenen, Schmand, Lindeboom, & Harska, 1992) (r = 0.06), which makes it unlikely that education was a confounder to the effects of age on the experimental data^54^. Data were collected in the laboratories of the Donders Center for Cognition, Radboud University (Nijmegen, Netherlands). Data were collected in accordance with the Declaration of Helsinki on ethical principles and with national and institutional ethical guidelines. Before participation, participants gave their written informed consent. They were compensated for their participation with giftcards of a value of €10 per hour. Completing the eye-tracking experiment lasted 15–20 min. For completing the whole test battery, including breaks, participants spend 2–3.5 h in the laboratory in one or two sessions. The study was ethically approved by the Ethical Review Board of the Faculty of Social Sciences of the Radboud University (ECSW2017-2306-520).

**Table 1:** Sample characteristics.

	Mean (SD), Range
Age	41.44 (20.09), 19–85
Sex	27F/18 M (60% F)
Education	17.47 (3.56), 10–25
Verbal IQ (NLV)	84.91 (9.13), 54–98
Memory recall (15WT)	7.72 (3.69), 0–15
Recognition (15WT)	27.26 (2.85), 18–30
Visuo-motor speed (LDST)	40 (8.69), 24–73

Demographic information and neuropsychological test results for the sample. Education is reported in years, including years of higher education and/or vocational training. *Test abbreviations and units*: 15WT: 15 words test (15 woorden test)^55^, number of correctly recalled/recognized words; NLV: Nederlandse Leestest voor Volwassenen (Dutch Adult Reading Test)^56^, raw score based on number of correctly pronounced words; LDST: Letter Digit Substitution Test^57^, time to complete test sheet in seconds.

### Task and stimuli

**Fig. 1:**
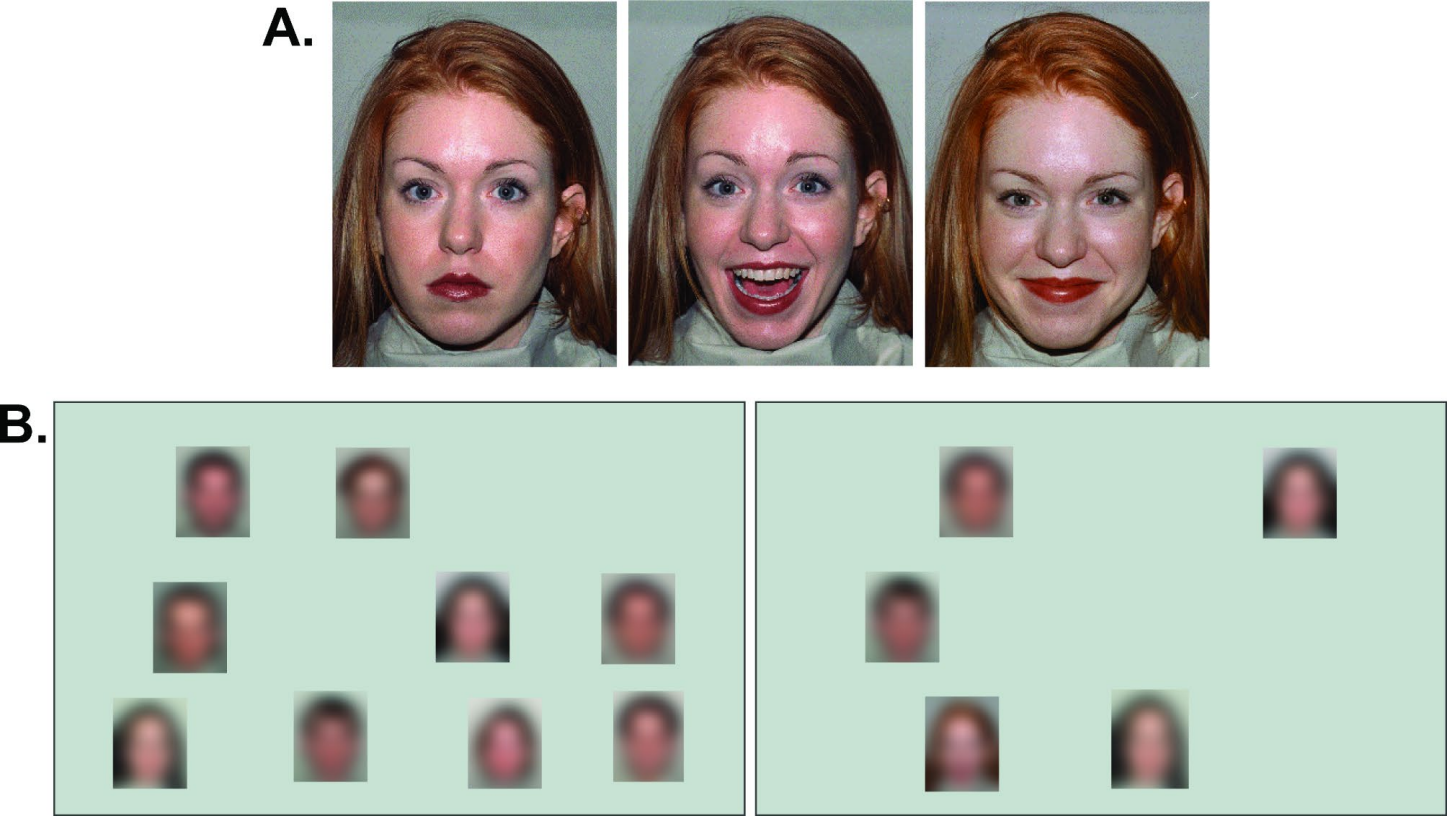
Stimuli and trial displays. (**A**) Examples of the neutral face (left), the dissimilar target (center), and the similar target (right). (**B**) Examples of search displays in target-present trials with a set size of 10 stimuli and target-similar distractor (left) and with a set size of 5 stimuli and target-dissimilar distractor (right). Images are blurred due to due copyright of the original stimulus material.

Participants’ task was to indicate whether or not a “happy” (smiling) target face was present in the search display that otherwise contained faces with neutral expressions (see Fig. 1). Before each search block (see 2.4), the ten happy target faces of the respective target category (similar to the distractors, i.e. smiling without visible teeth, or dissimilar to the distractors, i.e. smiling with visible teeth) and the ten neutral distractor faces were displayed side by side with the written phrase “Search these faces…” positioned over the possible targets and the written phrase “… among these neutral faces” (in Dutch). The participant then started the search block themselves with a keypress. Images in the search display did not overlap. The search display was shown until a manual response was registered. Responses were given with a right-hand (index or middle finger) key press. In case of an error, a short beep was issued as feedback. Instructions emphasized both speed and accuracy.

Face stimuli were drawn from the NimStim stimulus set^58^, similar to studies by Horstmann and colleagues^16,17^. We used five female models and five male models from the set. For each of these models, we used a neutral face and two variants of friendly faces, one smiling with an open mouth and visible teeth (dissimilar to the distractors), and one smiling with a closed mouth (similar to the distractors, see Fig. 1A). The neutral distractor faces all had a closed mouth. Thus, a total of 30 pictures of faces were used. Each color picture subtended 77 × 99 pixels (1.5° × 2.2°), and was coded as a bitmap with a color depth of 8 bits (see Fig. 1 for an example of the three expressions that were used from each model).

Also similar to, and as reported in the studies by Horstmann and colleagues^16,17^, search displays consisted of five or ten face stimuli. The stimuli were presented at randomly selected locations from an imaginary grid of 21 (7 horizontal × 3 vertical) locations. Figure 1B shows two examples of target-present trials, one with a similar target (left panel) and one with a dissimilar target (right panel). The central position of the grid contained the fixation marker in the pre-stimulus display and was excluded from use as a stimulus position. The fixation marker was a black disk with a small white center (standard fixation stimulus for the SR-1000 eye tracker, a bulls-eye, which is a white 4 px circle centered on a black 16 px circle). Center to center distances of the grid position were 200 pixels (4.4° of visual angle) horizontally and vertically. On each trial, each stimulus position was randomly jittered horizontally and vertically, by − 10, − 5, 0, 5, or 10 pixels.

### Apparatus

Stimuli were presented on a 24-inch computer screen (Benq XL2420Z, resolution 1920 × 1080) at a viewing distance of 78 cm. As in previous studies^16^, we used a video-based tower-mounted eye tracker (EyeLink 1000, SR Research, Ontario, Canada). Eye movements were recorded with a sampling rate of 1000 Hz. Participants’ placed their heads in a chin and forehead rest. The right eye was monitored. Prior to the experiment, the eye tracker was calibrated using a 9-point calibration. The experiment, including stimulus presentation and response recording, was programmed with Experiment Builder 2.4.1 (SR Research, Ontario, Canada) and eye tracking data were preprocessed with the Data Viewer 4.2.1 (SR Research, Ontario, Canada).

### Design

The experiment consisted of eight experimental blocks with 20 trials each. In four of the eight blocks, participants searched for a similar target and in the other four blocks, participants searched for a dissimilar target. Blocks with similar and dissimilar targets occurred alternating, and half of the participants started with a similar target block, so that block order was counterbalanced. Each block contained ten target-present and ten target-absent trials. Importantly, target-absent trials in the similar and dissimilar target conditions were structurally identical: both displayed five or ten distractors. In target-present trials, one of the distractors was randomly chosen to be replaced with a target faces of the respective target category in that block (similar vs. dissimilar targets). The target in each trial was selected pseudo-randomly, so that each individual target face appeared equally often in each block (see also^17^, for a similar design). Before the experiment proper, participants completed a 20-trial practice block, showing the two types of targets (similar vs. dissimilar), trial types (present vs. absent), set sizes (5 vs. 10), and some of the possible target stimuli. Responses during the practice block were not recorded.

### Data preprocessing

Data processing was similar to, and as reported in a previous study^19^. Raw eye position data were parsed by a speed threshold (30°/s) and an acceleration threshold (8,000°/s^2^) for saccade detection (the eye tracker software’s standard experimental settings). Rectangular areas of interest (AOI) of 101 × 130 pixels were defined that enclosed the stimulus shapes for all possible stimulus position, whether occupied by a stimulus or not; outlier fixations were assigned to the nearest of the 21 AOI. AOI not occupied by stimuli were eliminated from the data file before analysis proper, leaving 5 AOI for the set size 5 condition and 10 AOI for the set size 10 condition. Then, we derived four variables for analysis. First, each stimulus was classified as being fixated (in the AOI) within a given trial or not. Dwell time was measured if a stimulus was fixated. Dwell time was calculated as the sum of all fixation durations over the first continuous series of fixations on that stimulus. Thus, dwell time might often be based on the duration of a single fixation. However, in case a participant fixated the same stimulus multiple times during the first continuous visit, for example to foveate different regions of the face or because the first saccade was not optimally placed to foveate the most informative region, the time of the additional fixations was added. Please note that the gaze duration of possible revisits were dismissed in the calculation of dwell time. This was done to avoid confounding the measures of dwelling and revisiting. We separately recorded whether a stimulus was visited only once, or whether it was revisited, that is, selected repeatedly during a trial after the first continuous run of fixations. A fixation was scored as a revisit if (a) the stimulus had been fixated before and (b) the last fixation of that stimulus was interrupted by at least one off-stimulus fixation.

The basic variables of our analysis were trial statistics (i.e., statistics for each trial) of Dwelling, Skipping, Revisiting, and RT. *Dwelling* is defined as the average dwell time in a given trial. *Skipping* is defined as the proportion of stimuli that had not been fixated at all in a trial. *Revisiting* is defined as the proportion of stimuli that had been revisited. Finally, RT was measured as the time elapsed between display onset and key press of a correct answer.

Raw data for the measures of time (i.e., correct RT, dwell times, and stimulus selection latencies) were log-transformed and filtered for outliers prior to statistical analyses. We classified data points as outliers when they exceeded the mean of their respective condition (target presence x similarity x set size) by two standard deviations or more. Furthermore, a bottom cutoff of 300 ms was applied for RTs and of 40 ms for dwell times. Outlier analysis was performed for each participant individually. In total, this led to the removal of 124 RTs (1%), and 1156 dwell times (3%). See^18^ for a similar procedure of outlier correction.

### Data analysis

Data analyses were performed using R^59^. First, we performed descriptive analyses to illustrate differences in the behavioral (RT, and proportion of correct answers, PC) and eye-movement measures (skipping rate, dwell time, revisiting rate) between the manipulated search conditions (target presence, similarity, and set size), across the entire sample (N = 45). We also computed bivariate correlations between RT, age, skipping rate, dwell time, revisiting rate, similarity, and set size, on the level of trials, for the target absent trials. Here, correlations between binary variables (similarity and set size) and continuous variables (RT, dwell time, skipping rate, revisiting rate) indicate that the level of the former affects the latter, similar to main effects in a factorial design.

Notably, the correlations among multiple predictors complicate a direct interpretation of the bivariate relationships of the variables. Thus, in our main analyses, we used multiple linear mixed effect regression models to assess the effects of age on dwelling, skipping, and revisiting, and to assess whether age and all three distractor rejection mechanisms predict search times with random intercepts for the 45 subjects to quantify the independent effects of predictor variables while holding the other effects constant. Metrical variables (RT, skipping rate, dwell time, revisiting rate) were z-transformed prior to analyses in order to make regression coefficients comparable. The analyses were based on individual trials, which were 3495 observations per variable in total. Generally, we interpreted t-values exceeding a value of ± 1.96 as significant. Note, however, that due to the large number of observations, even small coefficients are often significantly different from zero and coefficients should be evaluated with respect to their size, not their statistical significance (< 0.10 very small, 0.10–0.29 small effect, 0.30–0.49 medium effect, 0.50 < large effect)^60^. In the correlations and multiple linear regressions, we focused on target-absent trials, because only in these trials, skipping, dwelling, and revisiting can be observed independently of the processes that may lead to the selection of the target in target-present trials.

First, we assessed whether age predicts skipping rates, dwell time, and revisiting rates, in similar relative to dissimilar target search, and in search through a display set size of 10 relative to 5 distractors. Each of the eye-tracking measures, in target-absent trials, were thus regressed on age, similarity, and set size as predictors, to quantify the effect of age on skipping, dwelling, and revisiting, and potential interactions of age with search difficulty, manipulated by target-distractor similarity and set size.

Second, we regressed trial-based RT on age and the trial statistics for distractor skipping, dwelling, and revisiting, to indicate whether age and the three underlying mechanisms are independent predictors for search times. For the experimental factor target-distractor similarity, low similarity was dummy-coded as zero and high similarity was coded as one. For the experimental factor set size, set size 5 was dummy-coded as zero and set size 10 was coded as one. In all regression analyses, the *variance inflation factor* (*VIF*) was used to guard against the possibility of multicollinearity among the predictor variables. For all models, the *VIF* was acceptable with tolerances (i.e., 1/*VIF*) within > 0.70 (well above the critical tolerance level of 0.1).

## Results

### Descriptives

**Fig. 2:**
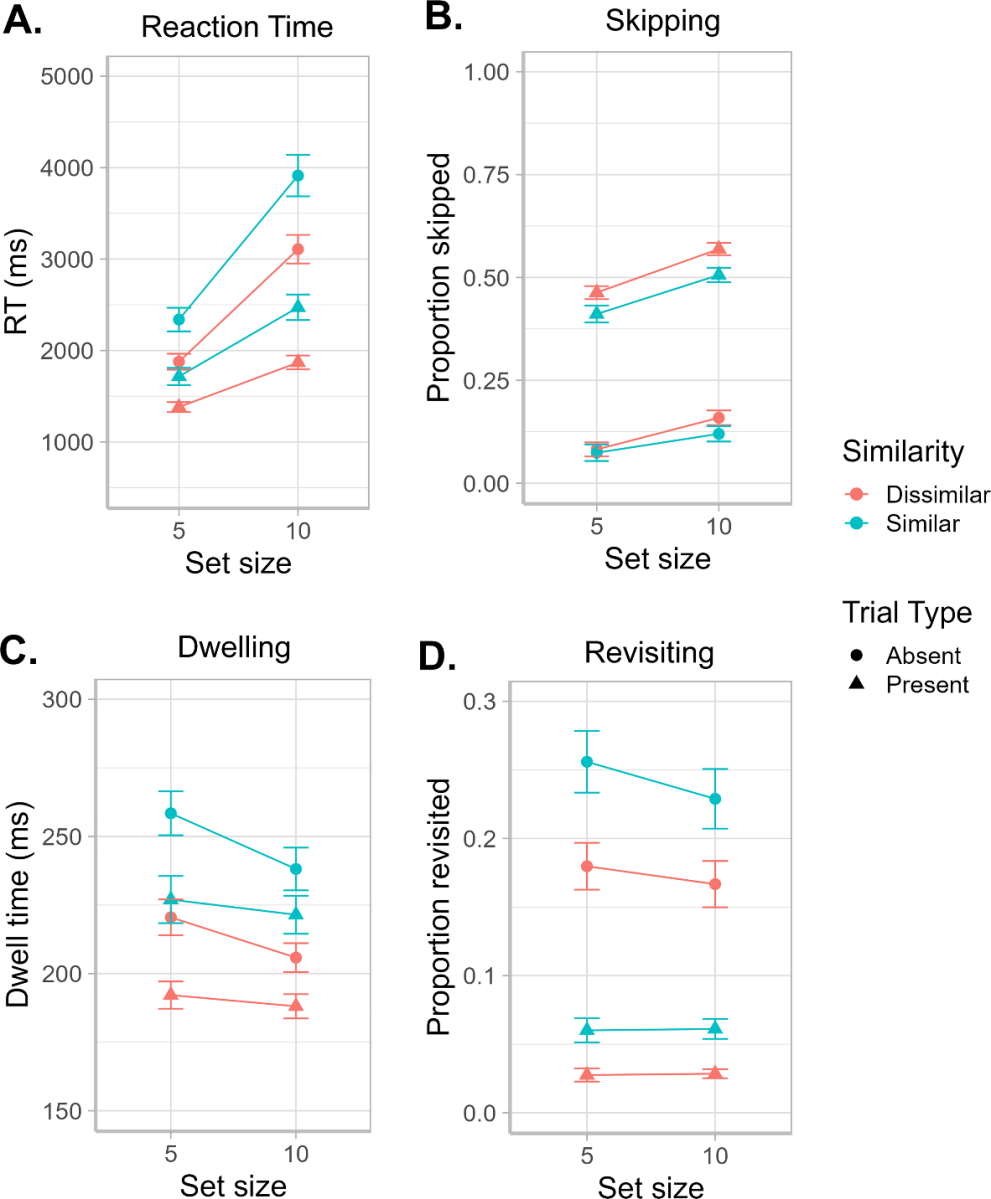
Descriptives of reaction time and eye-movement data. Means and standard error of the mean are plotted for (**A**) reaction time, (**B**) rates of skipping, (**C**) dwell time, and (**D**) rates of revisiting distractors, in each of the search conditions. The error bars show the standard error of the mean.

Means of RT, skipping rates, dwell time, and revisiting rates are plotted for the different search conditions in Fig. 2. The values show the expected effects of target presence, target-distractor similarity, and visual set size on RT and eye-movement measures^16,17,19^. Means of proportion of correct answers (PC) are reported for the different search conditions in Table 2.

**Table 2:** Error rates.

Target presence	Similarity	Set size	Mean	SD
Absent	Dissimilar	5	0.99	0.02
		10	1.00	0.01
	Similar	5	1.00	0.02
		10	0.99	0.04
Present	Dissimilar	5	0.94	0.06
		10	0.89	0.08
	Similar	5	0.92	0.07
		10	0.90	0.11

Mean and standard deviation of the proportion of correct answers (PC) in each of the search conditions.

**Fig. 3:**
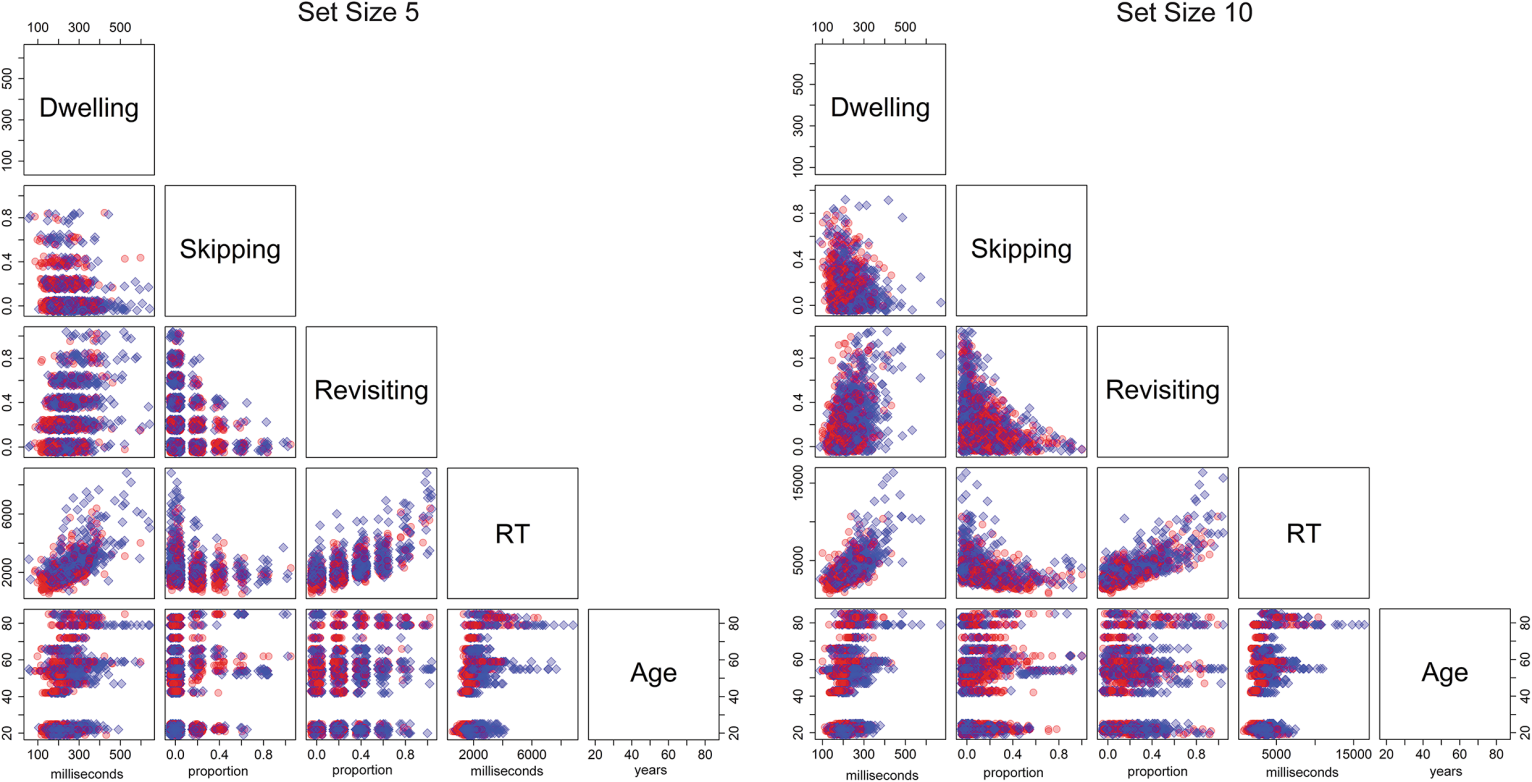
Correlations. Bivariate relationship between age (in years), trial search times (RT, in ms), revisiting rates (Revisiting), skipping rates (Skipping), and dwell times (Dwelling, in ms), for distractors in target absent trials per participant and trial in the set size 5 condition (**A**) and set size 10 condition (**B**). Target-dissimilar and target-similar distractors are presented as red circles and blue diamonds, respectively. Note that the data look more discrete for set size 5 than set size 10, as the values for skipping and revisiting could only assume a multiple of 0.2.

Table 3 presents the bivariate correlations between RT, age, skipping rate, dwell time, revisiting rate, similarity, and set size, on the level of trials, for the target absent trials. Figure 3 presents the corresponding scatterplots. Each dot represents one trial, where blue dots correspond to trials from similar blocks and red dots correspond to trials from dissimilar blocks. The linear relationships indicate that longer RT are associated with more revisiting, more dwelling, and less skipping. Aging is associated with longer RT, more skipping, more revisiting, and longer dwelling. Furthermore, RT increases with set size and similarity. Skipping increases with set size. Revisiting increases with similarity. Dwelling increases with similarity and somewhat decreases with set size.

**Table 3:** Correlations.

	RT	Skipping	Revisiting	Dwelling
Skipping	− .10			
Revisiting	.59	− .30		
Dwelling	.50	− .15	.36	
Similarity	.21	− .08	.16	.26
Set size	.48	.19	− .05	− .14
Age	.37	.11	.21	.35

Bivariate correlations between trial search times (RT), age, revisiting rate, skipping rate, dwell time, similarity, and set size, on the level of trials, are reported for the target absent trials. All correlations were significant.

### Regression analyses

#### Age effects on skipping, dwelling, and revisiting

First, we assessed whether age predicts skipping rates, dwell time, and revisiting rates, in similar relative to dissimilar target search, and in search through a display set size of 10 relative to 5 distractors. The results are displayed in Table 4. Plots illustrating the main effects and interactions with age can further be found in the online supplementary material https://osf.io/vc67p/.

**Table 4:** Results of regressing dwelling, skipping, and revisiting, on age, similarity, and set size.

	Skipping			Dwelling			Revisiting		
	b	sd(b)	t	b	sd(b)	t	b	sd(b)	t
Intercept	− 0.09	0.10	− 0.93	− 0.14	0.08	− 1.62	− 0.12	0.08	− 1.58
Age	0.11	0.10	1.13	**0.36**	0.08	4.30	**0.20**	0.08	2.58
Similarity	− **0.14**	0.03	− 5.48	**0.54**	0.02	23.08	**0.32**	0.03	11.58
Set size	**0.37**	0.03	14.91	− **0.27**	0.02	− 11.53	− **0.09**	0.03	− 3.32
Age x Similarity	**0.10**	0.03	3.98	**0.05**	0.02	2.18	0.03	0.03	1.18
Age x Set size	− **0.07**	0.03	− 2.65	− **0.08**	0.02	− 3.23	− 0.04	0.03	− 1.35

Linear multilevel regression of age on dwell times, skipping rates, revisiting rates, and similarity and set size as fixed effects and random intercepts for participants, in target absent trials. Note that multilevel models do not have clear-cut degrees of freedom for t-values. With a high number of observations, t-distributions converge with the standard normal distribution and we interpreted empirical t-values >  ± 1.96 as significant. B-values of significant effects are displayed in bold.

The multiple regression with *skipping* as the criterion revealed a small effect of similarity and a medium effect of set size. Fewer distractors were skipped if they were similar to targets, and more distractors were skipped if the set size was higher. The effect of age was small and not significant. However, (very) small, but significant age x similarity and age x set size interactions were found. The negative similarity effect decreased with age, and the set size effect decreased somewhat with age.

The multiple regression with *dwelling* as the criterion revealed small to medium effects of age, similarity, and set size. Dwell times increased with age and with similarity, but decreased with set size. The age x similarity and age x set size interaction effects were very small, with the similarity effect somewhat and the (negative) set size effect significantly increasing with age.

The multiple regression with *revisiting* as criterion revealed a small effect of age, a medium effect of similarity, and a small effect of set size. Older participants revisited distractors more often than younger adults. More distractors were revisited when the target was similar and somewhat fewer distractors were revisited if the set size was larger. The age x similarity and age x set size interactions were very small and not significant.

#### Effects of age, dwelling, skipping, and revisiting on RT

Secondly, we regressed trial-based RT on age and the trial statistics for distractor dwelling, skipping, and revisiting, to indicate whether age and the three distractor rejection processes were independent predictors for search times. The regression yielded a good fit with the data with a marginal R^2^ = 0.72. Table 5 shows the results for predicting RT in target-absent trials.

**Table 5:** Results of regressing search times (RT) on age, dwelling, skipping, and revisiting.

	b	sd(b)	t
Intercept	− 0.62	0.05	− 13.5
Skipping	− 0.11	0.01	− 10.7
Dwelling	0.25	0.01	24.1
Revisiting	0.36	0.01	38.5
Age	0.21	0.04	4.8
Similarity	0.17	0.02	10.9
Set size	1.09	0.01	74.4

Linear multilevel regression of age, dwelling, skipping, revisiting, and similarity and set size as fixed effects and random intercepts for participants, in target absent trials. All effects were significant.

All variables predicted RT significantly. The largest effect was found for set size, which means that RT substantially increases with higher numbers of distractors. Similarity only had a small effect on RT, which implies that RT increase slightly if the target is more similar to the distractors. Furthermore, RT increased with age, longer dwelling, and more revisiting, while skipping somewhat decreased RT. Note that these effect reflect the unique contributions, independently from the effects of age on dwelling, skipping, and revisiting reported above.

## Discussion

**Fig. 4:**
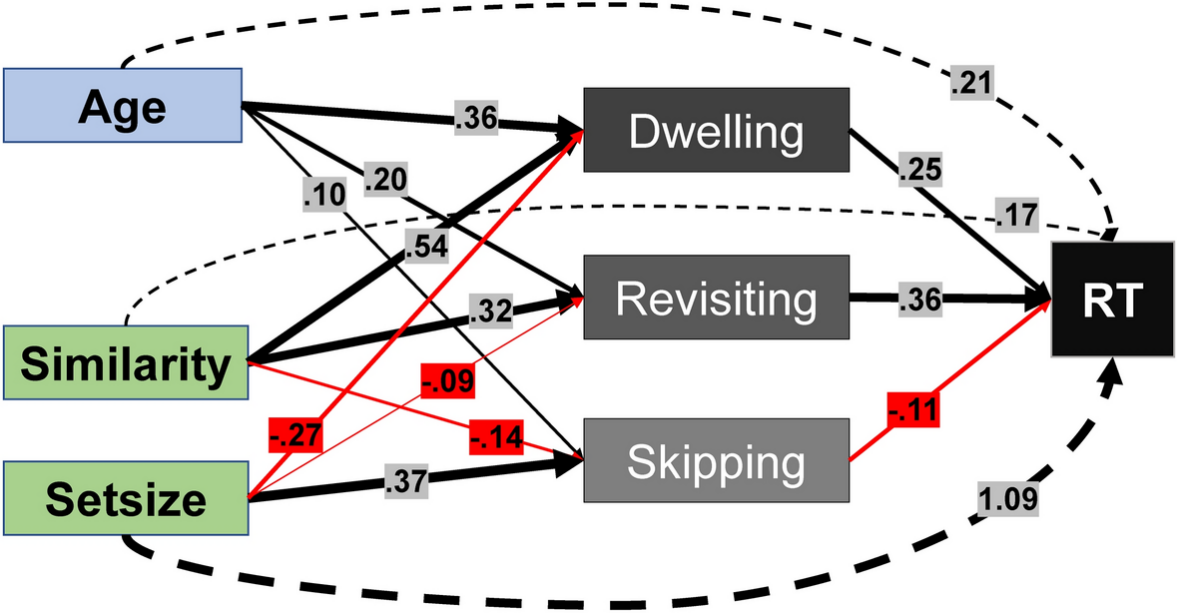
Path model showing the relationships between age, similarity, and set size, and dwelling, skipping, revisiting, and between all variables and search times (RT). Positive relationships are indicated by black arrows and positive beta weights and negative relationships are indicated by red arrows and negative beta weights. The weight of the lines represents the strength of the relationship (also indicated by the beta weights).

To better understand the origins of the well-known age-related slowing in visual search, the present study examined age differences in three distractor rejection processes that independently contribute to search times: the skipping of distractors, the dwelling on distractors, and the revisiting of distractors. To observe distractor rejection processes independently of target selection processes, we analyzed target-absent trials. An overview of the relationships between age, search conditions, eye-movement measures, and search times is depicted in the path model in Fig. 4. Replicating previous research, search times increased with age and with search difficulty (set size and target distractor similarity). As expected, aging was associated with longer dwelling and more revisits. Contrary to our hypotheses, however, skipping rates were affected little by aging, and contributed less to search times than dwelling and revisiting. Finally, age also predicted search times independently, beyond the age effect on dwelling and revisiting.

### Effects of age and search difficulty on distractor skipping, dwelling, and revisiting

First, contrary to our hypotheses, we found that *skipping rates* did not decline with age; numerically, skipping rates even increased with age. Our original reasoning was based on the assumption that older participants may adopt a more conservative decision rule and tend to search exhaustively in target-absent trials, while younger adults are more risky and may abort search more easily when they are not successful^51,61^. Our results, however, indicate that the decision rule contributing to skipping did not generally vary with age.

As in previous studies, participants skipped fewer distractors in displays with high target-distractor similarity and skipped more distractors at larger set sizes^16,18–20^. The similarity effect on skipping can be explained by guidance-based accounts, which assume that participants increase their quitting threshold if target-distractor similarity is high, due to the lower signal-to-noise ratio in the attention-guiding activation map^62^. As a consequence, they skip fewer stimuli if targets and distractors look more alike.

Adaptation of the quitting threshold based on guidance cannot account for the (negative) set size effect on skipping rates. Rather, reduced skipping at larger set sizes could be explained by more stimuli being processed in parallel within a single fixation in denser displays. The retinal region in which a target can be reliably identified among distractors with a single fixation has been referred to as the Functional View Field (FVF)^44^. As more stimuli fall within the FVF in the larger set size condition, more stimuli can be skipped compared to sparser displays in the small set size condition. The effects of similarity and set size on skipping rates were both slightly reduced with age. However, although statistically significant, the small effect size of the interactions with age limits their practical relevance and meaningful interpretation.

Second, in line with our hypotheses, we found that *dwelling* on distractors increased with age. The age effect on dwelling implies that older, relative to younger, observers spend more time processing a stimulus before it is classified as a distractor. Prolonged stimulus processing might be a consequence of slower visual processing or visual sensory decline in older age^36,63–65^. Alternatively, or additionally, longer dwell times (which equal the sum of fixation durations on a stimulus) may result from longer fixation durations, which have been linked to age-related decline in attentional disengagement from distractors^14,50,66,67^. Furthermore, older adults may dwell longer because they need more time for preparing the saccade to the next stimulus^68^.

Regardless of age, participants dwelled longer on distractors if target-distractor similarity was high. The similarity effect on dwelling is in accordance with previous results, and supports the claim that dwell times depend on the difficulty of target discrimination, rather than being constant across visual search conditions^19,20,31^. Furthermore, we found that dwell times decreased with set size and that this (negative) set size effect on dwell times increased with age. Longer dwelling in sparser displays may result from the longer preparation of saccades to the next selected item when the spatial separation of stimuli is larger^68^. As older adults need more time for saccade preparation^69^, the effect on dwell times may scale up with age.

Third, the *rate of revisiting* distractors increased with age. Similarly, previous studies reported that older, as compared to younger, adults made more fixations^50,66^ and revisited previously inspected areas in the search display more often^7,70^. Revisiting has been related to the (in)efficiency of IOR in longer and difficult search tasks. However, IOR was shown to be preserved in older adults^46^ and, thus, may not account for the age-related increase in revisiting we find in the present study. Rather, more revisiting in older age may reflect a failure or faster decay of visuo-spatial memory for previously visited locations^71^. The capacity for storing visited locations is strongly limited^44,72,73^ and known to further decline with age^36,49,74^. Thus, age-related memory loss is a plausible cause for more revisiting of previously attended items we observe in older age.

Apart from the age effect, and in line with previous results, we found that revisiting increased with target-distractor similarity and decreased with set size^16,17,19,20^. The similarity effect on revisiting could also be explained by memory failure: If target-distractor similarity is high, search is more difficult and takes longer, and memory fades at longer intervals. Memory loss, however, cannot explain the (negative) set size effect on revisiting. Memory overflow and, thus, forgetting, should be higher at larger set sizes; however, we observed *less* revisiting in displays with more distractors. Possibly, this effect may simply reflect a random component in saccade target selection: the chance to hit the same distractor twice is just smaller in large set sizes, for pure statistical reasons.

### Contribution of age, skipping, dwelling, and revisiting to RT

Assuming that skipping, dwelling, and revisiting determine visual search time, age differences in these variables should also contribute to the well-known age-related slowing in visual search^2,51^. Our results show that age primarily affects dwelling and revisiting, which, in turn, influenced the final search times. This suggests that longer search times in older age resulted, at least partially, from the effects of age on dwelling and revisiting. By contrast, the contribution of skipping to search times was rather small and age affected skipping inversely to the effect of skipping on search times. Accordingly, less skipping cannot explain any of the age-related slowing in the present task (see Fig. 4). Furthermore, age predicted RT also independently of dwelling, and revisiting. This implies that other processes, not captured in the reported eye-tracking measures, contributed to generalized age-related slowing in search times. Likely, age-related psychomotor slowing affected processes before or after the actual search, such as an increase in perceptual threshold^75^, response preparation, and response execution^23^.

Regardless of age, and as expected, search times were longer under more difficult search conditions with high target-distractor similarity and more distractors in the display^21,27^. However, different mechanisms appear to cause the RT costs of these two difficulty manipulations. Similarity had a rather small independent effect on search times, presumably because the influence was primarily explained through its effect on dwelling and revisiting. By contrast, the relationship between set size and skipping, dwelling, and revisiting was inverse to the relationship between each of the distractor rejection processes and RT (see Fig. 4). This is an interesting observation: While a strong effect of set size on RT is, of course, expected a priori^27^, parts of this effect appear to be absorbed or dampened by distractor rejection processes we measured by eye-tracking.

### Limitations and future directions

It should be noted that the skipping rates in the present experiment were generally relatively low (10–20%, see Fig. 2), which is expected when using a difficult, inefficient search task. The overall low skipping rates may also explain why skipping has a relatively minor contribution to individual trial differences and age-related increases in RT. From the present results, we may therefore not infer that skipping is unaffected by aging in general, but limit this conclusion to difficult searches. The age effect on skipping, and its contribution to RT, may thus be reexamined under conditions with stronger target guidance that lead to higher skipping rates of distractors overall.

While our sample size was larger than previous studies in younger adults, and the trial-based analyses with high numbers of observations were well-powered, the power to detect main effects or interactions with age is limited to the detection of effects of a moderate size. Furthermore, the sample size does not allow including further covariates in the regression models to control for confounding factors, such as education (although education was unrelated to age in our sample). In addition, the age distribution within the sample was not even, in particular, missing participants in the age range of 30–40 years.

## Conclusions

Our findings suggest that multiple distinct, capacity limited processes contribute to age-related decline in difficult visual search^36^. Age-related slowing of visual search was associated with longer dwelling on and more revisiting of distractors. Longer dwelling in older age likely results from reduced visual processing speed, while more revisiting might be explained by age-related decline in visuo-spatial memory. By contrast, skipping rates did not decrease with age. Thus, the decision thresholds based on target guidance appear to be largely unaffected by aging, and do not contribute to older adult’s longer search time in difficult visual search.

## Data Availability

The data and R script to analyse the results of this manuscript are available on the Open Science Framework at: https://osf.io/7w65m/ The data can be found under DOI 10 .17605/OSF.IO/AVYN6.
